# PET-MR Guided, Pre-targeted delivery to HER2(+) Breast Cancer Model

**DOI:** 10.21203/rs.3.rs-3974001/v1

**Published:** 2024-02-28

**Authors:** Ge Si, Sudath Hapuarachchige, Wojciech G. Lesniak, Dmitri Artemov

**Affiliations:** Department of Chemical and Biomolecular Engineering, The Johns Hopkins University, 3400 N. Charles Street, Baltimore, MD 21218, USA; The Russell H. Morgan Department of Radiology and Radiological Science, The Johns Hopkins University School of Medicine, 720 Rutland Ave, Baltimore, MD 21205, USA; Department of Oncology, The Sidney Kimmel Comprehensive Cancer Center, The Johns Hopkins University School of Medicine, 401 N. Broadway, Baltimore, MD 21287, USA; The Russell H. Morgan Department of Radiology and Radiological Science, The Johns Hopkins University School of Medicine, 720 Rutland Ave, Baltimore, MD 21205, USA; The Russell H. Morgan Department of Radiology and Radiological Science, The Johns Hopkins University School of Medicine, 720 Rutland Ave, Baltimore, MD 21205, USA; Department of Oncology, The Sidney Kimmel Comprehensive Cancer Center, The Johns Hopkins University School of Medicine, 401 N. Broadway, Baltimore, MD 21287, USA; The Russell H. Morgan Department of Radiology and Radiological Science, The Johns Hopkins University School of Medicine, 720 Rutland Ave, Baltimore, MD 21205, USA

**Keywords:** uSPIO, PET-MR, Pretargeting, Nanocarrier Delivery, Cancer imaging, HER2(+) breast cancer, biorthogonal click chemistry

## Abstract

**Purpose:**

HER2(+) metastatic breast cancer (mBC) is one of the most aggressive and lethal cancer types among females. While initially effective, targeted therapeutic approaches with trastuzumab and pertuzumab antibodies and antibody-drug conjugates (ADC) lack long-term efficacy against HER2(+) mBC and can cause severe systemic toxicity due to off-target effects. Therefore, the development of novel targeted delivery platforms that minimize toxicity and increase therapeutic efficacy is critical to the treatment of HER2(+) breast cancer (BC). A pretargeting delivery platform can minimize the non-specific accumulation and off-target toxicity caused by traditional one-step delivery method by separating the single delivery step into a pre-targeting step with high-affinity biomarker binding ligand followed by the subsequent delivery step of therapeutic component with fast clearance. Each delivery component is functionalized with bioorthogonal reactive groups that quickly react *in situ*, forming cross-linked clusters on the cell surface, which facilitates rapid internalization and intracellular delivery of therapeutics.

**Procedures:**

We have successfully developed a click chemistry-based pretargeting platform for HER2(+) BC enabling PET-MR image guidance for reduced radiation dose, high sensitivity, and good soft tissue contrast. Radiolabeled trastuzumab and superparamagnetic iron-oxide carriers (uSPIO) were selected as pretargeting and delivery components, respectively. HER2(+) BT-474 cell line and corresponding xenografts were used for *in vitro* and *in vivo* studies.

**Results:**

An enhanced tumor accumulation as well as tumor-to-organ accumulation ratio was observed in pretargeted mice up to 24 h post uSPIO injection. A 40% local T_1_ decrease in the pretargeted mice tumor was observed within 4 h, and an overall 15% T_1_ drop was retained for 24 h post uSPIO injection.

**Conclusions:**

Prolonged tumor retention and increased tumor-to-organ accumulation ratio provided a solid foundation for pretargeted image-guided delivery approach for *in vivo* applications.

## Introduction

Breast cancer is the most prevalent cancer type in the female population, and HER2(+) cancer constitutes 20%–30% of total breast cancer cases.[1, 2] HER2 receptor overexpression correlates with rapid tumor progression, high grade, and poor prognosis.[3] The discovery of HER2 overexpression has motivated researchers to investigate therapeutic agents that target HER2 receptors. Humanized monoclonal antibody trastuzumab (Tz) has high binding affinity to the HER2 receptor and possesses anti-proliferation activity on HER2(+) cell lines.[4] Combined treatment with Tz and chemotherapeutics and, more recently, with Tz antibody-drug conjugates (ADC), such as ado-trastuzumab (T-DM1), and trastuzumab deruxtecan (T-DXd) have shown an efficient tumor growth inhibition and have become most successful ADCs, used for treatment of patients with HER2(+) BC.[5, 6] However, ADCs show long circulation times and inefficient internalization, inhibiting the delivery of drugs into the tumor cells.[6–8] Due to off-site accumulation and premature release of the cytotoxic cargo in circulation, these single-step delivery systems may cause systemic toxicity that leads to a narrow therapeutic window. Therefore, implementing a pretargeting step is critical to minimize the off-target effects of the therapeutic carrier delivery component.

Pretargeting image-guided delivery platforms have been intensely studied and steadily evolved for the past three decades.[9, 10] The pretargeting strategy circumvents the premature release of cytotoxic cargo from long circulating carriers and non-specific accumulation of the traditional one-step delivery method by separating it into a pre-targeting of the receptor followed by a subsequent delivery of therapeutic and/or imaging component.[11, 12] In the pre-targeting step, a high-affinity ligand specifically targets the unique tumor microenvironment or receptors overexpressed on the surface of cancer cells without internalization. In the second step, a molecule or nanocarrier component with an optimized drug load and pharmacokinetics is administrated and selectively accumulates in locations, where the pretargeting bioligand is present. Components of each delivery step are conjugated *in situ* via bioorthogonal reactive groups. If a cell-surface target is bound by delivery components with multiple reactive groups, a web structure is formed on the cell surface that not only improves binding efficiency, but also promotes internalization, which facilitates the delivery of therapeutics.[13, 14]

Successful implementations of pretargeting strategy have been reported in both preclinical and clinical studies.[15–18] However, a PET-MR simultaneous imaging platform-based pretargeting strategy highlighting a T_1_ MR contrast has not been reported. For fast in situ conjugation between the PET pretargeting component and MR active delivery component, we have used bio-orthogonal click chemistry as a backbone of our pretargeting delivery system.[19] Trans-Cyclooctene (TCO) and tetrazine (Tt) inverse-electron-demand Diels − Alder reaction is one of the fastest click chemistry reaction with a reaction rate of *k* = 10^3^ ~ 10^6^ *M*^−1^ *s* ^−1^.[20, 21] The system uses commercially available TCO and Tt linkers that we have successfully implemented in pretargeting strategy.[22, 23]

In this study, we synthesized a panel of distinct MR reporters based on the new superparamagnetic iron oxide nanoparticles, uSPIO construct, each bearing different reactive groups and/or uorophores and used for control and experiment groups. We first confirmed the *in vitro* binding of uSPIO-Tt-Rhodamine to trastuzumab-TCO pretargeted BT-474 cells. Then, the pretargeting strategy was studied *in vivo* in mice bearing HER2 + tumors and both the pretargeting component trastuzumab-TCO-^89^Zr and delivery component uSPIO-tetrazine were monitored with PET-MR imaging. Colocalization of PET and MR signal was only detected in the tumor region of pretargeted mice, in agreement with the initial hypothesis of our pretargeting model.

## Materials and Methods

PEG linker Azido-PEG23-amine was purchased from BroadPharm. Oleic acid-capped uSPIO (5 nm core size, 25 mg/mL in CHCl_3_) was purchased from Ocean NanoTech. Rhodamine-NHS and tetrazine-NHS were purchased from Thermo Fisher Scientific. Trastuzumab was provided by Genentech, Inc. All other chemicals, solvents, and materials of analytical grade were purchased from MilliporeSigma. Zirconium-89 oxalate, ^89^Zr(C_2_O_4_)^2−^ (*t*_1/2_ = 78.4 h) was obtained from Washington University (St. Louis, MO).

Amicon^®^ Ultra Centrifugal MWCO 30kDa filter units were purchased from MilliporeSigma, Inc. and used for ultra-centrifugal ltration.

### Synthesis of uSPIO-Rhodamine (Scheme 1a).

Hydrophilic uSPIO with surface primary amine groups was synthesized as described in our previous report.[24] For fluorescence labeling, NHS-Rhodamine (Rhod) (0.185 mg, 25 molar equiv. to uSPIO) was dissolved in 10 *μ*L of dry DMSO, then added to 2 mL of 1 mg/mL uSPIO aqueous solution at pH = 7.4 and mixed in the dark under room temperature for 1.5 h. The crude mixture was passed through a PD-10 column (equilibrated by pH = 7.4 1×PBS) to eliminate any unbound rhodamine. The collected eluent with the product was further purified and concentrated by 1×PBS using a 30 kDa MWCO centrifugal filter unit until no more pink color was present in the ltrate. The final uSPIO-Rhod product was stored at a concentration of 1mg/mL in 1×PBS at 4°C.

### Synthesis of uSPIO-Tetrazine (Scheme 1b).

Tetrazine-PEG_4_-NHS (0.2 mg, 30 molar equiv. to uSPIO) (Tt) was dissolved in 10 *μ*L of anhydrous DMSO and then added to a uSPIO solution (2 mL of 1.0 mg/mL in 1×PBS). The reaction was stirred in the dark under room temperature for 1.5 h. The crude mixture was passed through a PD-10 desalting column to eliminate unreacted Tt. The product was further purified and concentrated using a 30 kDa MWCO centrifugal filter unit until no more pink color in the ltrate was observed. The final uSPIO-Tt product was stored at a 1.0 mg/mL concentration in 1×PBS at 4°C.

### Synthesis of uSPIO-Tt-Rhodamine (Scheme 1c).

NHS-Rhodamine (0.185 mg, 25 molar equiv. to uSPIO) was dissolved in 10 *μ*L of anhydrous DMSO, then added to 2 mL of 1 mg/mL uSPIO-Tt in 1×PBS. The reaction was stirred in the dark at room temperature for 1.5 h. The crude mixture was passed through a PD-10 desalting column to eliminate excess Rhod. The collected solution was further purified and concentrated as described above. The final uSPIO-Tt-Rhod product was stored at a 1.0 mg/mL concentration in 1×PBS at 4°C.

### Synthesis of Tz-TCO-AF488 (Scheme 1d).

Tz-TCO-AF488 was synthesized according to our previously published protocol.[11, 22, 23] Brie y, Trastuzumab (500 μ L of 10 mg/mL in 1×PBS) was treated with TCO-NHS ester (30 moles equiv. in 10–20 μ L of dry DMSO) for 1 h with gentle stirring. Samples were purified by ultracentrifugation followed by SEC. The degree of functionalization was determined based on the change of molecular weight measured by MALDI-TOF. The resulting Tz(TCO)_6_ (500 μL of 10 mg/mL in 1×PBS) was treated with NHS-AlexaFluor 488 (10 moles equiv. dissolved in 10 μL of anhydrous DMSO) and stirred for 1 h in the dark. The degree of labeling (DOL) of the fluorophore was maintained at 2–4. The products were purified by ultra ltration followed by size-exclusion column chromatography.

**Tz-TCO-DFO** and **Tz-TCO-DFO[**^**89**^**Zr]** were synthesized and characterized according to our published protocol.[23] Radiolabeling of Tz-TCO-DFO with ^89^Zr was performed using a previously reported procedure.[23] 100 μL of 1.0 M oxalic acid containing 3.0 mCi of ^89^Zr was mixed with 50 μL of 2.0 M Na_2_CO_3_ and incubated for 3 min at room temperature. Then, 200 μL of 0.5 M HEPES (pH ~ 7) containing 1 mg of Tz-TCO-DFO was added. The resulting mixture was incubated for 1 h at room temperature with gentle rocking. The radiolabeling efficiency was measured using ITLC (Pall Life Science) and 20 mM citric acid as an eluent. Tz-TCO-DFO [^89^Zr] was purified on a Zeba spin column (0.5 mL MWCO: 7 kDa), preequilibrated with sterile 1×PBS. The concentration of antibody in Tz-TCO-[DFO]^89^Zr was determined based on the absorbance at 280 nm measured on a Nanodrop 2000 UV–vis spectrophotometer (Thermo Fisher Scientific). Applied radiolabeling procedure provided Tz-TCO-DFO[^89^Zr] with approximately 99% radiochemical purity and 2.8 ± 0.2 mCi/mg specific activity. For further studies, the specific activity of Tz-TCO-DFO[^89^Zr] was adjusted to 37 mBq (1.0 mCi)/mg using unlabeled Tz-TCO-DFO.

### Characterization of uSPIO constructs.

Core sizes of uSPIO constructs are characterized using Hitachi 7600 TEM. At least 150 uSPIO particles in one continuous TEM ROI were measured for an average core size using ImageJ. Hydrodynamic diameters (HD) of uSPIO constructs were measured by Malvern Zetasizer Nano ZS90 in pH = 7.4 1×PBS solution, with 3 measurements and 10 runs per measurement for each construct. Intensity distribution was used for HD analysis.

### In Vitro binding.

BT-474 cells were grown in 46-X medium supported by 10% Fetal Bovine Serum, 100 units/mL penicillin, and 100 units/mL Penicillin-Streptomycin (Pen-Strep). Cells were seeded into 4-well chamber slides at Passage 3, at a seeding density of 50,000 cells/chamber. After 2 days of cell growth, the medium was removed from all chambers and 0.25 mL of 35 *μg*/*mL* Tz-TCO-AF488 in medium was added to chambers 1, 2, and 3 of each chamber slide, 0.25 mL of medium was added to chamber 4 of each slide as controls. Chambers were incubated on ice for 30 min. After incubation, all chambers were washed with sterile 1×PBS. Then 0.25mL of 35 *μg*/*mL* uSPIO-Tt-Rhod in medium was added to chambers 1 and 2 of each chamber slide, 0.25mL 35 *μg*/*mL* uSPIO-Rhod in medium was added to chamber 3 of each chamber All chamber slides were incubated in the incubator maintained at 37°C and 5% CO_2._ Chamber slides were incubated for 45 min and 4 hours, respectively (Supporting Information S3).

After the incubation period, medium was removed, and slides were washed with sterile 1×PBS. Cells were xed with 4% Paraformaldehyde (PFA) solution for 15 min in the dark at room temperature, aspirated and washed with 1×PBS. 2.5 *μg*/*mL* Hoescht 33342 solution was used for nuclei staining in the dark at room temperature for 8 min, aspirated and washed with 1×PBS. The chamber dividers were removed, and slides were mounted with cover slips using mounting agent with photobleaching inhibitor. Mounted slides were allowed to solidify overnight before confocal microscopy.

### Confocal microscopy.

Cells were imaged using a Zeiss LSM700 Single-point, laser scanning confocal microscope equipped with 405, 488, and 639 nm lasers used to excite blue nuclei, green Trastuzumab and red uSPIO, respectively. A 20 nm gap between the excitation and detection wavelengths was used for fluorescence detection in all channels. A dual-line scanning method was used, and alignment was enforced before each scanning. The pinhole size of 1 AU (Airy Unit) was set for each color channel under the same magnification. Confocal images were taken using a 43x oil immersion objective. Image processing was done with ImageJ. Individual channels were used for individual analysis and then merged for display.

### In vivoTumor and Mouse models.

BT-474 breast cancer cells (ATCC) were grown in 46-X media supplemented with 10% Fetal bovine serum (Sigma-Aldrich), 100 units/mL of penicillin and streptomycin for inoculation of 6–8 weeks old athymic Nu/Nu female mice (Charles River Laboratories). Estrogen pellet 17β-estradiol (0.72mg, 90-day release, Innovative Research of America) was implanted in the subdermal area on the back of each mouse 24 h prior to inoculation. BT-474 cells. (2×10^6^ cells in 50% of matrigel/media) were inoculated into the 2nd mammary fat-pad of the mouse. Mice were used for imaging when tumors reached 200–300 mm^3^. All animal procedures were performed in accordance with the United States Public Health Service Policy on the Humane Care and Use of Laboratory Animals and approved by the Johns Hopkins University Animal Care and Use Committee.

### Two step, pretargeted delivery and imaging.

**Figure 1:**
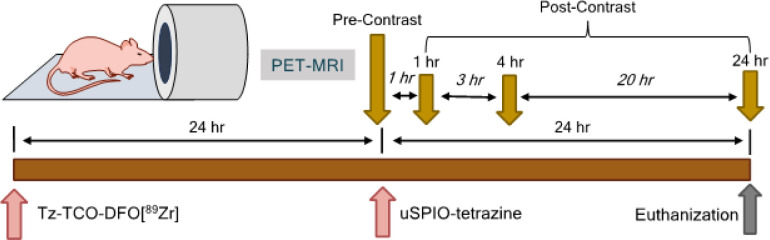
Timeline of PET-MR Imaging. Tz-TCO-[DFO]^89^Zr was injected and mice were imaged 24 h post injection to verify tumor specific accumulation. After pre-contrast MR images were collected, uSPIO-tetrazine was injected. Post-contrast MR images were acquired at 1, 4, and 24 h after uSPIO-injection.

PET-MR imaging was performed to visualize biodistribution of both the pretargeting component and the delivery component in mice bearing BT-474 tumors (Fig. 1). Mice were randomly split into 2 groups: pretargeted and non-pretargeted groups (n = 3). Pretargeted mice were intravenously injected with 13.875 MBq (0.375 mCi) of Tz-TCO-DFO[^89^Zr] containing 0.2 mg of Tz-TOC-DFO. Non-pretargeted group received same volume of saline. PET-MR images were collected for each mouse 24 h post Tz-TCO-DFO [^89^Zr] injection. These images served as a baseline MR. After these images were obtained, 280 μg uSPIO-Tt was administered to all mice of by the tail vein injection. Prior to the injection, uSPIO-Tt was sterilized by ltration through 0.22 μm PVDF lters. PET-MR images were recorded for each mouse at 1, 4 and 24 hours post uSPIO-Tt injection.

The field of view (FOV) of axial T_1_ MR images were chosen to cover the entire chest and abdominal region of the animal using 20 slices with slice thickness of 1.5 mm. T_1_ maps were acquired using RARE fast-spin echo sequence, with TE set at 13 ms and varying TR at 650, 1000, 1500, 2000, 4000 and 8000 ms, and in plane resolution of 0.25 mm. T_1_ maps were reconstructed with IDL and/or Python software developed in our lab. T_2_-contrast MR images were used for co-registration with PET scans. The FOV of coronal T_2_ MR images covered the entire body as well. T_2_ contrast images were acquired with RARE fast spin-echo sequence, with T_E_ = 32 ms and T_R_ = 3200 ms.

PET images were reconstructed with an on-board Bruker ParaVision software and processed with PMOD software (version 4.004) to fuse PET and MR coronal images. The standardized uptake value (SUV, %ID/cc) for the pretargeting component Tz-TCO-[DFO]^89^Zr in relevant tissues and tumors were analyzed with PMOD software, taken account of radioactive decay. PET-MR fused images and 3D PET-MR images were processed with ParaVision and PMOD software.

### Ex vivo biodistribution.

uSPIO uptake was measured from quantitative T_1_ analysis performed with the IDL programs and ImageJ image analysis software. uSPIO uptake was analyzed in the tumors, spleen, liver, and kidney. These organs were selected due to high iron concentrations detected by ICP-MS analysis in our previous study of untargeted uSPIO.[24] ImageJ was used to select ROIs for different organs in each slice. For each ROI, the surface area and the mean T_1_ values are recorded. After processing of all slices, an average T_1_ was calculated by weighing individual mean T_1_ with corresponding surface areas. All mice and all time points from each treatment group were analyzed.

### Statistical analysis

Statistical analysis done with JMP Pro 17 Software; P values of < 0.05 were considered significant.

## Results

### Nanomaterial Synthesis and Characterization.

uSPIO-based nanoparticles were conjugated with rhodamine, tetrazine and tetrazine + rhodamine. Their core sizes were 3.6 ± 0.2 nm, 3.6 ± 0.2 nm, and 3.4 ± 0.3 nm, respectively, with good uniformity (Supporting Information S1.1, S1.2). These sizes also match the unconjugated uSPIO size reported in our previous publication.[24] The hydrodynamic diameters (HD) of these uSPIO conjugates were 17.3 ters nm, 18.0 ters and 21.4 ters nm, respectively. All constructs have narrow HD distributions and are slightly larger than the unconjugated uSPIO, due to the conjugation of fluorophores and/ or click-reactive group (Supporting Information S2).

### In vitro Confocal Imaging.

**Figure 2:**
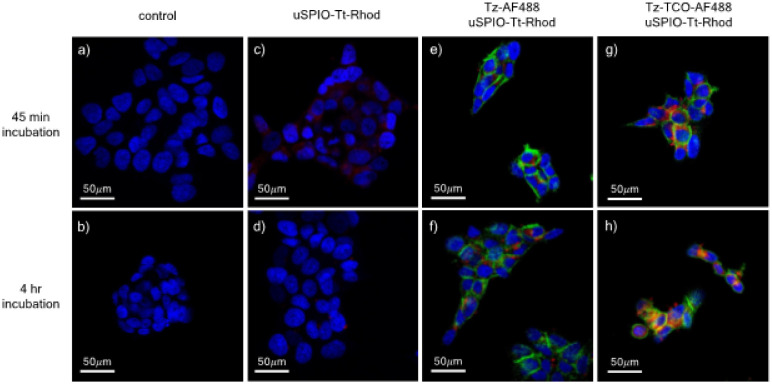
Confocal images of 4% PFA-fixed BT-474 cell in the chambers. All images were acquired with 40x objective. Nuclei are stained with Hoescht 33342 DNA dye (blue). a)-b) Control group: untreated with any agents. c)-d) non-pretargeted group, cells incubated only with uSPIO-Tt-Rhod (red) for 45 min and 4 h. e)-f) Pretargeted, none click-reactive group cells were first incubated with non-reactive Trastuzumab-AF488 (green) for 30 min, followed by the addition of uSPIO-Tt-Rhodamine, and 45 min and 4 h incubation. g)-h) Pretargeted, click-reactive group. Cells were rst treated with click reactive Trastuzumab-TCO-AF488, then treated with uSPIO-Tt-Rhodamine.

Processed images (Fig. 2) show that in non-pretargeted BT-474 cells treated with uSPIO-Tt-Rhod only, internalization is very low, suggesting minimal endocytosis of free uSPIO-Tt-Rhod. In both click-treated and none click-treated pretargeted groups, trastuzumab labeling was successful, as proved by the localization of green fluorescence on the cell surface. In the group with non-click-trastuzumab labeling, uSPIO was minimally internalized even after 4 h incubation, suggesting that click chemistry-driven ligation between components is necessary for uSPIO-Tt-Rhod membrane transport and endocytosis. In the cells preincubated with Tz-TCO-AF488, internalization of uSPIO-Tt-Rhod was observed starting from 45 min post uSPIO incubation. After 4 h incubation, more areas with higher signal intensity from rhodamine suggest that uSPIO-Tt-Rhod internalization increased with longer incubation time.

Individual and merged channels were examined in pretargeted groups (Figures S4.1 and S4.2). Figure S4.1 shows the channels for the 45 min incubation time point. Green channel (Tz labeling) signal readings are mostly from the cell surface, while some regions have initiated the caveolin-mediated endocytosis mediated by binding of Tz-TCO-AF488to HER2. In the click-reactive group, uSPIO-Tt-Rhod (red channel) were mainly located on the cell surface and some internalized due to click reaction between TCO and tetrazine, which has a very fast reaction kinetics. The yellow color seen on the merged image, mainly located near cell surface, suggested colocalization and reaction between the pretargeting component (Tz-TCO-AF488) and the delivery component (uSPIO-Tt-Rhod). In the none click-reactive group, uSPIO was not effectively internalized.

At 4 h incubation time point (Figure S4.2), significantly more Tz-TCO-AF488 pretargeting component was internalized compared to 45 min incubation time. In the none click-reactive group, uSPIO-Tt-Rhod did not internalize even after 4 h of incubation, suggesting limited efficiency of non-specific internalization pathways, such as pinocytosis or clathrin-mediated endocytosis in the delivery component internalization. This further confirms the effectiveness of the pretargeting strategy in enhanced *in vitro* internalization of nanocarriers.

### In vivo Study.

**Figure 3:**
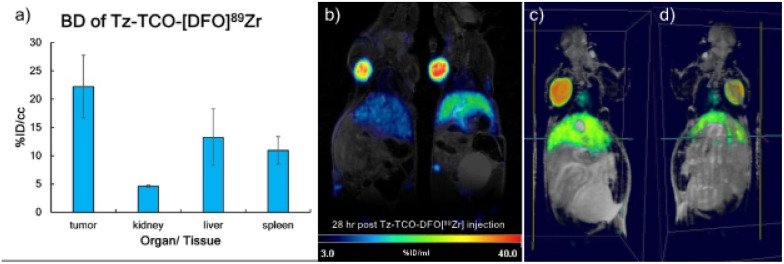
a) Biodistribution of Tz-TCO-DFO[^89^Zr], analyzed using PMOD software, normalized to the injection time point to take into consideration of radioactive decay. b) Co-registered PET-MRI images at 28 h after injection of Tz-TCO-DFO[^89^Zr]. Image fusion was done with PMOD software. c)-d) 3D PET-MR fused image. Volume-rendered images were generated with ParaVision Software.

Quantitative PET image analysis shows the highest radioactivity accumulation in the tumor region after 24 h of ^89^Zr injection (22%ID/cc in tumor), while less accumulations were detected in the liver and spleen (13 and 11%ID/cc, respectively, Figs. 3a). Tz-TCO-DFO[^89^Zr] retained in the tumor for at least 48 h (24 h before and 24 h after uSPIO injection), taking into account the radioactive decay (Fig. 3b). 3D fused PET-MR images produced using PMOD software are shown in Fig. 3c&d.

**Figure 4:**
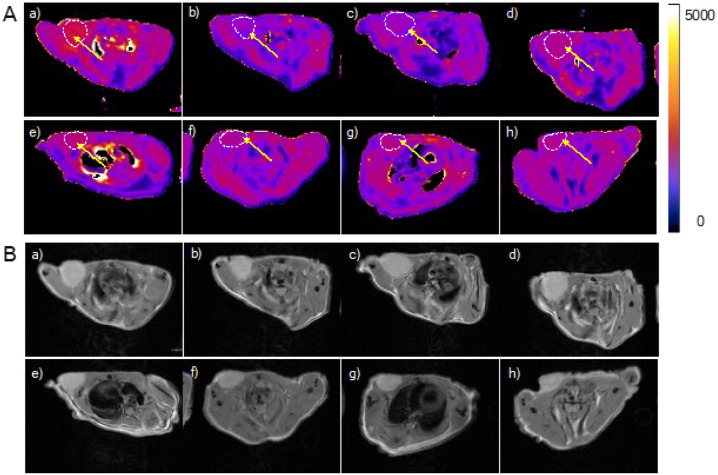
Quantitative T_1_ maps and corresponding original MR images used for quantitative relaxation mappings, before and after uSPIO-Tt injection. Figures a)-d): T_1_ mapping and original MRI images of the pretargeted mice group: a) pre-injection; b) 1 h post-injection; c) 4 h post-injection; d) 24 h post-injection. Figures e-h: T_1_ mapping and original MRI images of non-pretargeted mice group: e) pre-injection; f) 1 h post-injection; g) 4-h post-injection; h) 24 h post-injection. Tumor regions in mapped images are highlighted with white dotted curves. Original MRI images are proton-density weighted images, T_E_ = 13 ms, T_R_ = 8000 ms.

At 1 h post uSPIO-Tt injection, T_1_ map of the entire tumor ROI in the pretargeted group shows an overall 24% ± 3.8% percent decrease in T_1_ with some regions demonstrating T_1_ decrease over 40% (from 2810 ms to 2080 ms, Fig. 4, yellow arrow and Supporting Information S5), yet in the none pretargeted group tumor T_1_ drop of only 6% ± 0.5% was detected at 1 h post-contrast. At 4 h post-injection, T_1_ in the pretargeted group further decreased by 25% ± 2.8%the baseline value, with some regions having T_1_ reduced below 2000 ms. In contrast the T_1_ in the non-pretargeted group recovered to 95% of its original value at 4 h time point. At 24 h post-injection, T_1_ shortening is still persistent in the pretargeted group with an averaged 11% ± 0.3% reduction, while in the non-pretargeted group, T_1_ has fully recovered.

### Ex vivo Biodistribution Analysis.

**Figure 5:**
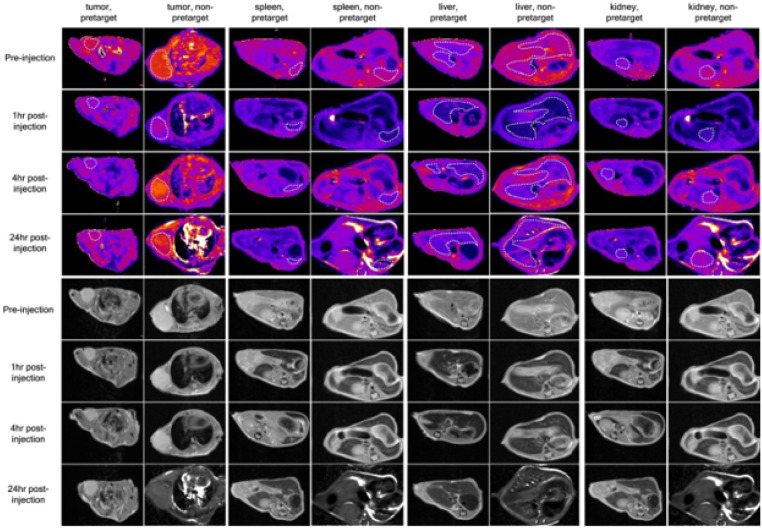
Quantitative biodistiibuticn analysis by T_1_ mapping. Color bar is in milliseconds. T_1_ for each organ of a mouse is calculated using equation: $T_{1}=\frac{\sum \left(SA\times T_{1,S}\right)}{\sum SA}$ where SA is the axial ROI surface area in a slice. T_1_.s is the average T_1_ value within tlie ROI of a slice. T_1_ is then weighed by the organ size (surface area) and averaged between all mice.

**Figure 6:**
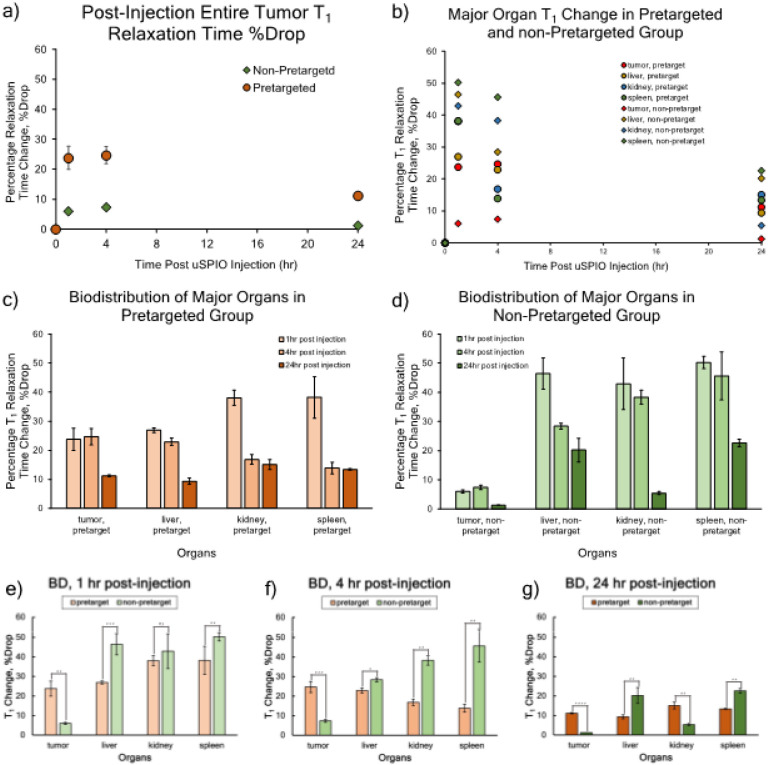
Percent decrease of T_1_ in organs from quantitative T_1_ analysis. a) Tumor T_1_ drop in both pretargeted and non-pretargeted groups; b) Major organs T_1_ drop in both groups. Pretargeting and non-pretargeting groups are distinguished by the marker shape. In both figures, round-shape marker stands for the pretargeted group, while diamond-shape marker stands for the non-pretargeted group. c) Biodistribution of uSPIO in the pretargeted group from T_1_ analysis. d) Biodistribution from T_1_ analysis in the non-pretargeted group. Error bars were calculated from results for 3 different animals in both groups. e)-g) Statistical Analysis between pretargeted and non-pretargeted groups, at all time points.

Representative T_1_ maps and corresponding original gray-scale MR image are shown in Fig. 5. Organs’ ROIs are highlighted with white dashed lines in the gure. The ROIs are depicted simply for representing the organs, the actual regions for T_1_ analysis were chosen carefully to avoid any false registration. Figure 6 shows quantitative results obtained from the analysis. In the pretargeted group (round-shape marker), the tumor demonstrated 4-fold higher T_1_ drop compared to the non-pretargeted group (diamond-shape marker) at 1 h (p < 0.01) as well as at 4 h post injection (p < 0.005) (Fig. 6a). At 24 h post injection, the difference between the two treatment groups was almost 10-fold (p < 0.0001). When cross comparing all major organs (Fig. 6b), non-pretargeted group experienced higher retention of uSPIO in all 3 major organs (liver, kidneys, and spleen) within 4 h post-injection than pretargeted group (p < 0.05, p < 0.01, p = 0.005), suggesting a non-specific uptake of nanoparticles. It is also worth noting that the tumor demonstrated the strongest T_1_ reduction compared to three major organs in the pretargeted group at 4 h post-injection, suggesting a target-specific accumulation of the nanoparticles by tumor in the pretargeting delivery approach. At 24 h the most significant T_1_ drop was detected in the liver and spleen in the non-pretargeted group. This agrees well with the ICP-MS analysis for uSPIO uptake in non-pretargeted mice shown in our previous study.[24]

## Discussions

Image guidance enables visualization of administrated components and hence becomes key to translational theranostics.[25, 26] In the pretargeting strategy, both components are conjugated with image probes utilizing orthogonal molecular imaging platforms. Combining PET imaging with MRI maximizes the information from the high sensitivity of a radioisotope and excellent spatial resolution and the enhanced soft tissue contrast of MRI.[27] An integrated bimodal imaging system such as PET-MRI can provide simultaneous image guidance of both pretargeting and delivery components. T_1_ contrast-enhancing MR reporters are favored for diagnostic imaging. Based on our previously developed sub-5nm hydrophilic uSPIO with enhanced T_1_ relaxivity and ample modification sites as well as our 7 Tesla PET-MRI system, we developed PET-MR image-guided pretargeting strategy featuring the rapid click-chemistry driven two-step delivery system.[24]

The *in vitro* confocal imaging showed that uSPIO-Tt-Rhod only internalizes when pretargeting components are present. This confirms the hypothesis that pretargeting is necessary and su cient for uSPIO-Tt-Rhod internalization. No cell death was seen in any of the treatment groups incubated with reagents and/or nanocarriers used during the labeling, as confirmed by comparing with cell morphology in the control group. At 4 h of incubation, more regions showed internalization in the click-reactive group compared to none click-reactive group, suggesting pretargeting might be a driving force for the enhanced ligand internalization. uSPIO-Tt-Rhod internalized and co-localized with Tz-TCO-AF488 only in the click-reactive group, suggesting the binding through TCO-tetrazine click chemistry reaction is efficient and stable.

Here we have assumed that the red signal from rhodamine corresponded to uSPIO, and the green signal corresponded to Alexa 488 labeled trastuzumab, and no free dissociated rhodamine or Alexa 488 was present at the time of imaging. This is a valid assumption for 2 reasons: 1) a total of 4 washing steps for uSPIO-Rhod and 6 steps for Tz-A488 have been performed throughout the binding study, this was also suggested by the clean background from all confocal images; 2) the untargeted uSPIO-Rhod group showed very low intensity and highly localized red signal, suggesting that the red fluorescence signal in the pretargeted group originated from the uSPIO-Tt-Rhod bound to Tz-TCO-AF488.

The *in vivo* coregistered PET-MR image verified the targeting efficiency and precision of the trastuzumab pretargeting. Tumor uptake data are in good agreement with our previous study, as well as the confocal study discussed earlier.[23] Internalized uSPIO starts disassembling after 2 h post injection and are mostly disintegrated at 24 h time point due to the presence of proteolytic enzymes and the acidic environment of late endosomes and lysosomes. In addition, compartmentalization of uSPIO in the intracellular organelles, such as late endosomes and lysosomes, can cause reduced T_1_ effects because of slow water exchange between cellular compartments and signal decay in T_2_ weighted images due to increased T_2_ susceptibility effects of concentrated and clustered nanoparticles.[28] Therefore, the actual amount of uSPIO accumulated in the tumor region can be higher than what was extrapolated from T_1-_weighted MR images. Nonetheless, our results still provide solid proof that the pretargeting strategy can enhance *in vivo* localization and have increased tumor retention of nanoparticles.

uSPIO-Tt showed a significant uptake in liver and spleen, most likely due to the presence of Kupffer cell macrophages that rapidly recognize, engulf, and retain nanoparticles in these organs. Of special interest is the uSPIO uptake in kidneys at 24 h post-injection. While organs in the non-pretargeted group are predicted to have higher non-specific accumulation, kidneys do not permanently retain nanoparticles and can eventually secrete them out even though the particle size is above the theoretical renal secretion threshold (14 nm vs 5 nm). However, this does align well with the ICP-MS analysis that showed minimal iron uptake in the kidney at 24 h. On the contrary, in the pretargeted group, the kidney uptake of uSPIO is more significant than other organs. However, mouse HER2 is not cross-reactive with trastuzumab, and a plausible explanation is that a trastuzumab-uSPIO nanoconjugates in circulation were clogging the renal filter, causing inefficiency in renal secretion. Bar graphs of uSPIO distribution for pretargeted vs non-pretargeted delivery show higher tumor uptake in the pretargeted group and overall higher non-specific uptake in the non-pretargeted group (Fig. 6c, 6d). These results strongly suggest the selective and prolonged retention of nanocarriers by the pretargeting delivery approach.

## Conclusions

We demonstrated that the pretargeting delivery strategy with Tz and uSPIO based nanocarrier increased site-specific accumulation of the carriers, prolonged the delivery component retention, and reduced off-target non-specific uptake. PET-MRI simultaneous dual-modality imaging enabled by the radiolabeled Tz-TCO-DFO[^89^Zr] and magnetic uSPIO-Tt with biocompatible surface coating with complimentary click-reactive groups allowed noninvasive monitoring of the delivery and biodistribution of both components. PET-MRI provides excellent sensitivity, spatial resolution and renders refined soft tissue details that not only enables accurate location of the site of action, but also allows quantitative analysis for pretargeting system. This study provided solid foundation for the use of ultrasmall superparamagnetic iron-oxide core nanoparticles as drug carriers for image-guided pretargeted delivery approach and its potential application in molecular imaging and theranostics.

## Abbreviations

ADC: Antibody Drug Conjugate
BC: Breast Cancer
HD: Hydrodynamic Diameter
mAb: Monoclonal Antibody
mBC: Metastatic Breast Cancer
Tz: Trastuzumab
uSPIO: ultrasmall Superparamagnetic Iron Oxide Nanoparticles

## References

[1] Siegel RL, Miller KD, Wagle NS, Jemal A (2023) Cancer statistics, 2023. CA Cancer J Clin 73:17–48. 10.3322/caac.21763 36633525

[2] Gutierrez C, Schiff R (2011) HER2: Biology, Detection, and Clinical Implications. Arch Pathol Lab Med 135:55–62. 10.5858/2010-0454-RAR.1 21204711 PMC3242418

[3] Waks AG, Winer EP (2019) Breast Cancer Treatment: A Review. JAMA 321:288–300. 10.1001/jama.2018.19323 30667505

[4] Gemmete JJ, Mukherji SK (2011) Trastuzumab (herceptin). AJNR Am J Neuroradiol 32:1373–4. 10.3174/ajnr.A2619 21816914 PMC7964332

[5] Lewis Phillips GD, Li G, Dugger DL, (2008) Targeting HER2-positive breast cancer with trastuzumab-DM1, an antibody-cytotoxic drug conjugate. Cancer Res 68:9280–90. 10.1158/0008-5472.CAN-08-1776. 19010901

[6] Cameron D, Piccart-Gebhart MJ, Gelber RD, (2017) 11 years’ follow-up of trastuzumab after adjuvant chemotherapy in HER2-positive early breast cancer: final analysis of the HERceptin Adjuvant (HERA) trial. Lancet 389:1195–1205. 10.1016/S0140-6736(16)32616-2 28215665 PMC5465633

[7] Swain SM, Shastry M, Hamilton E (2023) Targeting HER2-positive breast cancer: advances and future directions. Nat Rev Drug Discov 22:101–126. 10.1038/s41573-022-00579-0. 36344672 PMC9640784

[8] Kay C, Martínez-Pérez C, Meehan J, (2021) Current trends in the treatment of HR+/HER2+ breast cancer. Future Oncol 17:1665–1681. 10.2217/fon-2020-0504 33726508

[9] Halpern SE, Dillman RO (1987) Problems associated with radioimmunodetection and possibilities for future solutions. J Biol Response Mod 6:235–62 3298553

[10] Goodwin DA, Meares CF, David GF, (1986) Monoclonal antibodies as reversible equilibrium carriers of radiopharmaceuticals. Int J Rad Appl Instrum B 13:383–91. 10.1016/0883-2897(86)90015-2 3098706

[11] Hapuarachchige S, Zhu W, Kato Y, Artemov D (2014) Bioorthogonal, two-component delivery systems based on antibody and drug-loaded nanocarriers for enhanced internalization of nanotherapeutics. Biomaterials 35:2346–54. 10.1016/j.biomaterials.2013.11.075 24342725 PMC4332786

[12] Koo H, Lee S, Na JH, (2012) Bioorthogonal copper-free click chemistry in vivo for tumor-targeted delivery of nanoparticles. Angew Chem Int Ed Engl 51:11836–40. 10.1002/anie.201206703 23081905

[13] Zhu W, Okollie B, Artemov D (2007) Controlled internalization of Her-2/ neu receptors by cross-linking for targeted delivery. Cancer Biol Ther 6:1960–6. 10.4161/cbt.6.12.4979 18075296

[14] Martens TF, Remaut K, Demeester J, (2014) Intracellular delivery of nanomaterials: How to catch endosomal escape in the act. Nano Today 9:344–364. 10.1016/j.nantod.2014.04.011

[15] Jallinoja VIJ, Houghton JL (2021) Current Landscape in Clinical Pretargeted Radioimmunoimaging and Therapy. Journal of Nuclear Medicine 62:1200–1206. 10.2967/jnumed.120.260687 34016727 PMC8882889

[16] Hapuarachchige S, Artemov D (2020) Theranostic Pretargeting Drug Delivery and Imaging Platforms in Cancer Precision Medicine. Front Oncol 10:. 10.3389/fonc.2020.01131 PMC7387661 32793481

[17] Cheal SM, Chung SK, Vaughn BA, (2022) Pretargeting: A Path Forward for Radioimmunotherapy. Journal of Nuclear Medicine 63:1302–1315. 10.2967/jnumed.121.262186 36215514 PMC12079710

[18] Stéen EJL, Edem PE, Nørregaard K, (2018) Pretargeting in nuclear imaging and radionuclide therapy: Improving efficacy of theranostics and nanomedicines. Biomaterials 179:209–245. 10.1016/j.biomaterials.2018.06.021 30007471

[19] Rondon A, Degoul F (2020) Antibody Pretargeting Based on Bioorthogonal Click Chemistry for Cancer Imaging and Targeted Radionuclide Therapy. Bioconjug Chem 31:159–173. 10.1021/acs.bioconjchem.9b00761 31855602

[20] Emmetiere F, Irwin C, Viola-Villegas NT, (2013) (18)F-labeled-bioorthogonal liposomes for in vivo targeting. Bioconjug Chem 24:1784–9. 10.1021/bc400322h 24180480 PMC3903177

[21] Agarwal P, Beahm BJ, Shieh P, Bertozzi CR (2015) Systemic Fluorescence Imaging of Zebrafish Glycans with Bioorthogonal Chemistry. Angew Chem Int Ed Engl 54:11504–10. 10.1002/anie.201504249 26230529 PMC4694582

[22] Hapuarachchige S, Kato Y, Artemov D (2016) Bioorthogonal two-component drug delivery in HER2(+) breast cancer mouse models. Sci Rep 6:24298. 10.1038/srep24298 27068794 PMC4828666

[23] Hapuarachchige S, Si G, Huang CT, (2021) Dual-Modality PET–SPECT Image-Guided Pretargeting Delivery in HER2(+) Breast Cancer Models. Biomacromolecules 22:4606–4617. 10.1021/acs.biomac.1c00918 34704434 PMC8578463

[24] Si G, Hapuarachchige S, Artemov D (2022) Ultrasmall Superparamagnetic Iron Oxide Nanoparticles as Nanocarriers for Magnetic Resonance Imaging: Development and *In Vivo* Characterization. ACS Appl Nano Mater 5:9625–9632. 10.1021/acsanm.2c01835 37139481 PMC10153628

[25] Goldenberg DM, Chang C-H, Rossi EA, (2012) Pretargeted Molecular Imaging and Radioimmunotherapy. Theranostics 2:523–540. 10.7150/thno.3582 22737190 PMC3364558

[26] Pysz MA, Gambhir SS, Willmann JK (2010) Molecular imaging: current status and emerging strategies. Clin Radiol 65:500–16. 10.1016/j.crad.2010.03.011 20541650 PMC3150531

[27] Jauw YWS, O’Donoghue JA, Zijlstra JM, (2019) 89Zr-Immuno-PET: Toward a Noninvasive Clinical Tool to Measure Target Engagement of Therapeutic Antibodies In Vivo. J Nucl Med 60:1825–1832. 10.2967/jnumed.118.224568 31147401 PMC12079161

[28] Strijkers GJ, Hak S, Kok MB, (2009) Three-compartment T1 relaxation model for intracellular paramagnetic contrast agents. Magn Reson Med 61:1049–58. 10.1002/mrm.21919 19215042

